# Discovery of gene regulation mechanisms associated with uniconazole-induced cold tolerance in banana using integrated transcriptome and metabolome analysis

**DOI:** 10.1186/s12870-024-05027-2

**Published:** 2024-04-26

**Authors:** Liuyan Qin, Dandan Tian, Chenglin Guo, Liping Wei, Zhangfei He, Wei Zhou, Quyan Huang, Baoshen Li, Chaosheng Li, Mengyun Jiang

**Affiliations:** 1 Biotechnology Research Institute, Guangxi Academy of Agricultural Sciences, Nanning, 530007 China; 2 Institute of Plant Protection, Guangxi Academy of Agricultural Sciences, Nanning, 530007 China

**Keywords:** Banana, Uniconazole-induced, Cold tolerance, Metabolomic analysis, Transcriptomic analysis

## Abstract

**Background:**

The gibberellic acid (GA) inhibitor, uniconazole, is a plant growth regulator commonly used in banana cultivation to promote dwarfing but also enhances the cold resistance in plants. However, the mechanism of this induced cold resistance remains unclear.

**Results:**

We confirmed that uniconazole induced cold tolerance in bananas and that the activities of Superoxide dismutase and Peroxidase were increased in the uniconazole-treated bananas under cold stress when compared with the control groups. The transcriptome and metabolome of bananas treated with or without uniconazole were analyzed at different time points under cold stress. Compared to the control group, differentially expressed genes (DEGs) between adjacent time points in each uniconazole-treated group were enriched in plant-pathogen interactions, MAPK signaling pathway, and plant hormone signal transduction, which were closely related to stimulus-functional responses. Furthermore, the differentially abundant metabolites (DAMs) between adjacent time points were enriched in flavone and flavonol biosynthesis and linoleic acid metabolism pathways in the uniconazole-treated group than those in the control group. Temporal analysis of DEGs and DAMs in uniconazole-treated and control groups during cold stress showed that the different expression patterns in the two groups were enriched in the linoleic acid metabolism pathway. In addition to strengthening the antioxidant system and complex hormonal changes caused by GA inhibition, an enhanced linoleic acid metabolism can protect cell membrane stability, which may also be an important part of the cold resistance mechanism of uniconazole treatment in banana plants.

**Conclusions:**

This study provides information for understanding the mechanisms underlying inducible cold resistance in banana, which will benefit the production of this economically important crop.

**Supplementary Information:**

The online version contains supplementary material available at 10.1186/s12870-024-05027-2.

## Introduction

Banana (*Musa spp.*) is an economically important crop in global tropical and subtropical regions. Low temperature is one of the phenomena accompanying climate change and is one of the most important factors limiting the scale of banana cultivation to meet increasing food demand [1–3]. Bananas are thermophilic and their normal growth temperature ranges from 15℃ to 35℃. The low critical temperature for cultivated bananas is species-dependent and can range from 10 °C to 17℃, with the growth low critical temperature of most cultivated bananas in China is about 13℃ [4, 5]. Low-temperature stress during the growth period of fruit trees can cause physiological disorders within the plant that negatively impact fruit quality and lead to a decrease in their market value [3, 6].

Under cold stress, the photosynthesis rate of plants decreases and various forms of cell membrane damage occur [7, 8]. Cold tolerance in banana plants is enhanced by increasing the levels of compatible solutes such as proline, soluble carbohydrates, and phenolic compounds [1, 2]. Physiological processes, such as antioxidant capacity, photosynthesis, and plant hormone pathways, also tend to change in response to cold [5, 9–11].

Liu et al. reported that the sucrose content of cold tolerant wild banana ‘huanxi’ increased significantly when the temperature dropped and suggested that SNF1-related protein kinase catalytic subunit alpha 10, the central integrator of plant stress and energy signal transcription network, might be involved in the cold stress response by regulating sucrose biosynthesis [12]. Mitogen-activated protein kinases (MAPKs) are known to play important functions in cold stress responses of plants. MAPK5 has been shown to play a role in cold resistance of banana plants. In banana plants, the expression of *MAPK5* was upregulated under low temperature treatment, and the cold resistance of transgenic banana lines overexpressing MAPK5 was stronger than that of the control group [13]. MAPK3-inducer of C-repeat-binding factor expression 1 (ICE1)-Peroxidase P7 (POD P7) pathway and *plasma membrane intrinsic proteins 1;1* (*PIP1;1*) positively regulate the cold resistance in banana. MAPK3-ICE1-POD P7 pathway can significantly change the expression of cold-responsive genes and the oxidoreductase activity, *PIP1;1* can reduce ion leakage and malondialdehyde content, as well as increase proline, chlorophyll, soluble sugar, and abscisic acid (ABA) content [9, 14]. The overexpression of *dehydration-responsive element binding protein 1 F* (*DREB1F*) enhances banana resistance to cold stress by regulating the levels of protectant metabolites of soluble sugars and proline, activating the antioxidant system, and promoting jasmonate and ethylene synthesis [11].

Recently, high-throughput sequencing technology has been used to study cold resistance in bananas. Yang et al. (2015) identified common adaptive processes in bananas (a cold-sensitive group) and plantains (a cold-tolerant group) in response to cold stress through comparative transcriptomic studies [15]. Liu et al. (2018) analyzed the cold-responsive mRNA, lncRNAs, and miRNAs of a cold-resistant wild banana line, *Musa itinerans* under cold stress using RNA-seq. Cold stress conditions were found to alter or affect the pathways of photosynthesis, photosynthesis-antenna proteins, plant circadian rhythm, glutathione metabolism, starch and sucrose metabolism, and cutin/suberine/biosynthesis in banana, and miR172 was found to play an important coordinating role in response to cold stress [5, 16].

The gibberellic acid (GA) synthesis inhibitor, uniconazole, is commonly used as an effective plant growth regulator that promotes dwarfing in banana cultivation [17]. The use of uniconazole also enhances cold resistance by affecting some physiological processes in crops, such as mung bean (*Phaseolus radiatus*) and coix; however, the molecular mechanism by which uniconazole enhances plant cold resistance remains unclear [18, 19]. In this study, an integrative analysis of the transcriptomes and metabolomes of uniconazole-treated bananas grown under cold stress was performed to investigate the mechanisms by which uniconazole enhances cold resistance in plants. The results of this study provide a theoretical molecular strategy for improving cold resistance in bananas.

## Results

### Uniconazole induces cold tolerance in banana

**Fig. 1:**
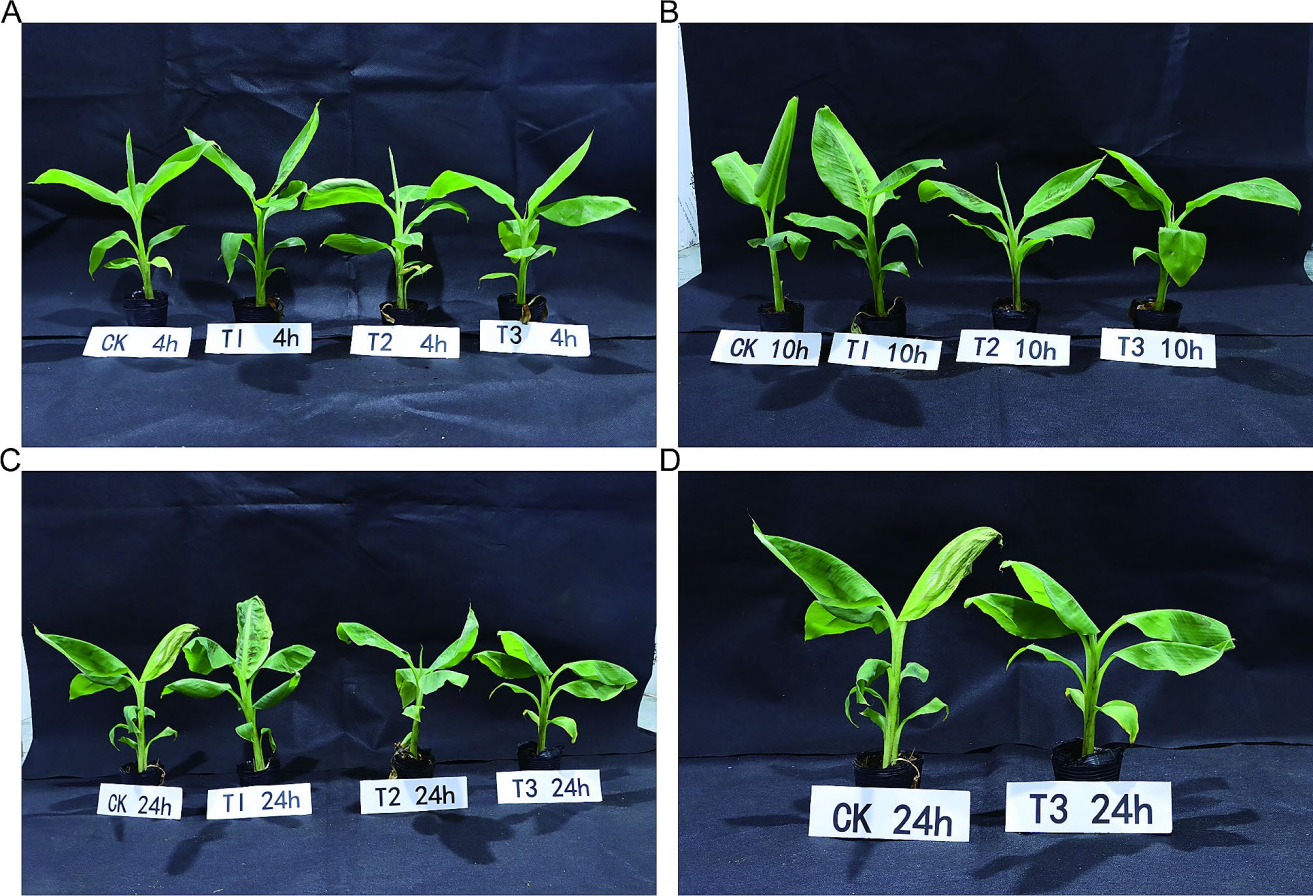
Characterization of uniconazole-induced cold tolerance in banana. Plant traits were treated with low temperature (4 ± 1℃) for 4 h (**A**), 10 h (**B**), and 24 h (**C**, **D**). T1, T2, and T3 indicate uniconazole treatments of 0.05 g, 0.1 g, and 0.2 g, respectively. CK indicates the water treatment that was used as the control

To confirm the cold tolerance effect of uniconazole in bananas, compared the induced traits of bananas treated with or without 0 g, 0.05 g, 0.1 g, and 0.2 g uniconazole and exposed to cold temperatures (Fig. 1). After 4 h at 4 ± 1℃, all leaves began to show black spots in four treatment groups (Fig. 1A). After 10 h at 4 ± 1℃, leaves showed significant wilting in the 0 g (CK), 0.05 g uniconazole (T1), and 0.1 g uniconazole (T2) treatment groups (Fig. 1B). After 24 h at 4 ± 1℃, the degree of leaf curl gradually decreased as the uniconazole concentration increased after 24 h at 4 ± 1℃ when compared with the CK group, especially plants treated with 0.2 g uniconazole (T3), which had fewer black spots on its leaves and a significant reduction in the number of curled banana leaves (Fig. 1C-D). These results suggested that uniconazole treatment can induce cold tolerance in banana.

### Physiological and biochemical changes in uniconazole-treated bananas

**Fig. 2:**
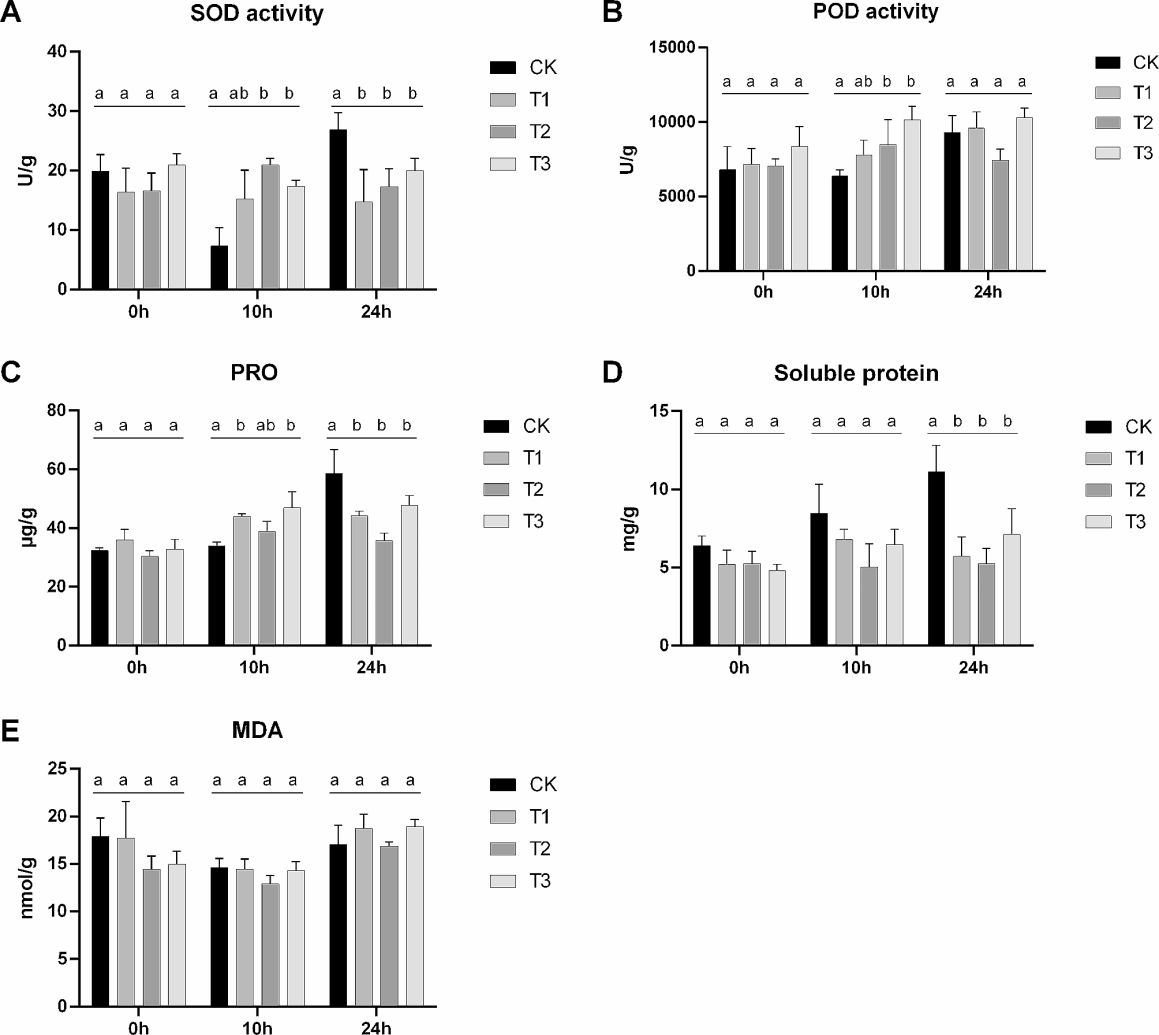
Physiological and biochemical changes induced by cold treatment. **A**: superoxide dismutase (SOD) activity; **B**: peroxidase (POD) activity; **C**: proline (PRO); **D**: soluble protein; **E**: malondialdehyde (MDA). T1, T2, and T3 indicate uniconazole treatments of 0.05 g, 0.1 g, and 0.2 g, respectively. CK indicates the water control treatment. Data are means ± SE of three replicates. Different letters over the points indicate a significant difference among different groups using Tukey’s test (*P* < 0.05)

To detect the physiological and biochemical changes caused by different concentrations of uniconazole in low-temperature conditions, superoxide dismutase (SOD) activity, peroxidase (POD) activity, and malondialdehyde (MDA), soluble protein, and proline (PRO) content were measured. The results showed that SOD and POD activities significantly increased after 10 h of low-temperature stress in T2 and T3 compared to those in CK (Fig. 2A and B), followed by a decrease in SOD activity after 24 h of cold temperature treatment (Fig. 2A). In addition, the content of PRO and soluble protein showed a significant decrease after 24 h of treatment with low-temperature stress in the three uniconazole treatment groups when compared with the CK (Fig. 2C and D), while the MDA level did not change in any of the groups (Fig. 2E). We chose 0.2 g uniconazole for further metabolomic and transcriptomic experiments based on to the best dwarfism traits induced in banana and the minimum effect on physiological and biochemical indices.

### Transcriptomic changes in response to uniconazole treatment in bananas

To evaluate gene expression changes in response to uniconazole treatment in bananas, we performed RNA-seq analysis of three biological replicate leaf samples treated with or without 0.2 g uniconazole per plant at three time points after low-temperature stress. The total clean reads in each sample ranged from 45,783,092 to 56,546,180 and were mapped to the reference genome at a high mapping rate (> 73.83; Table S1). Pearson’s correlation between replicates ranged from 0.89 to 1, suggesting that the transcriptome results were reliable and stable (Fig. S1).

**Fig. 3:**
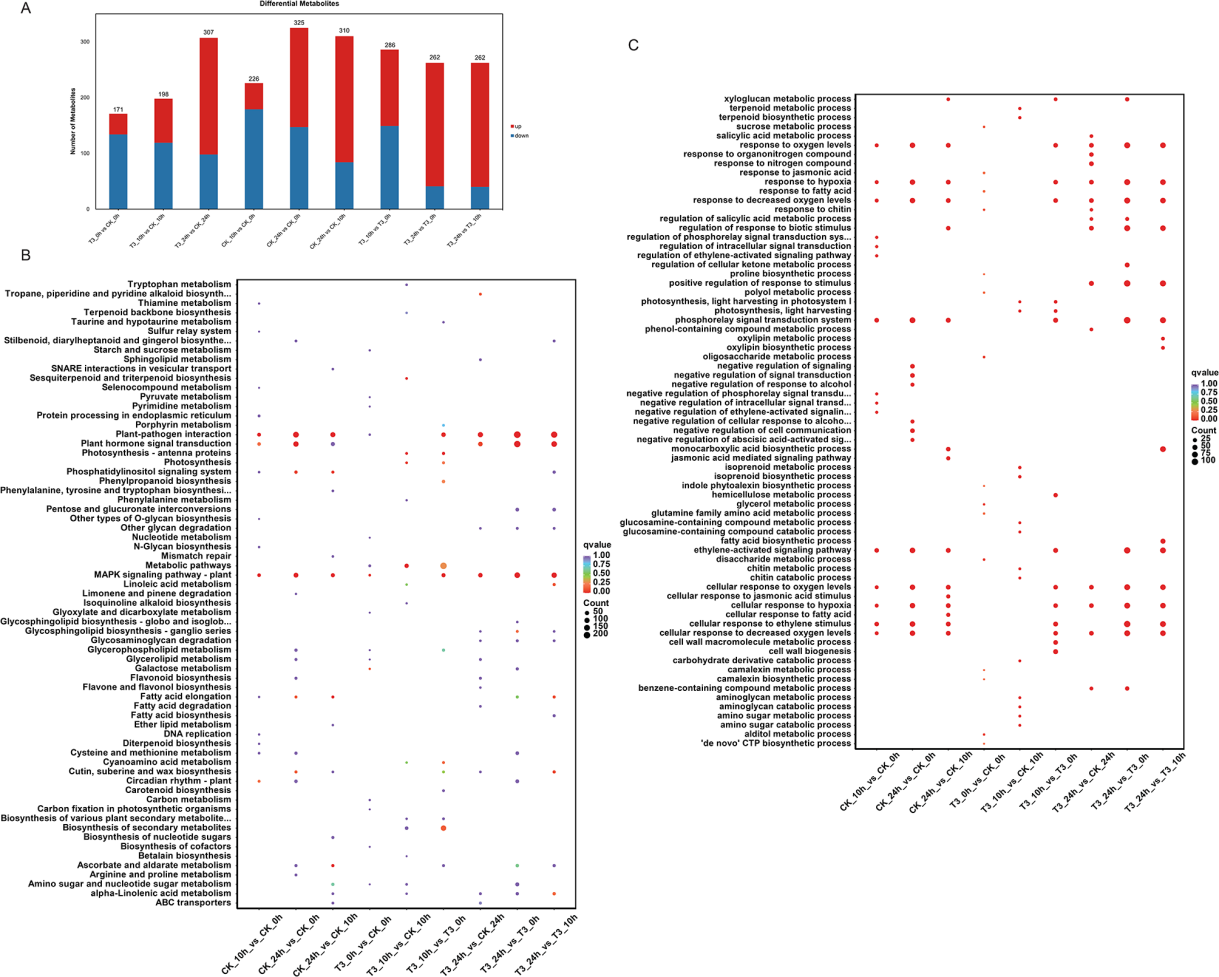
Differentially expressed genes (DEGs) between the uniconazole- and control-treated bananas induced by cold treatment. **A**: the numbers of DEGs between the different groups; **B**: KEGG pathway enrichment analysis of the DEGs; **C**: GO enrichment analysis of the DEGs. T3 indicate uniconazole treatments of 0.2 g. CK indicates the water control treatment

Differential expression analysis was performed for both uniconazole treatment and control samples collected different times during the low-temperature treatment (Fig. 3A). The number of upregulated genes was higher than that of downregulated genes for most comparisons, and the number of differentially expressed genes (DEGs) in the uniconazole treatment groups was much higher than those in the control at each time point (Fig. 3A). Among these DEGs, 140 and 1414 genes were upregulated, whereas 44 and 79 genes were downregulated in the T3_10h vs. CK_10h and T3_24h vs. CK_24h comparisons, respectively. Interestingly, when comparing 24 h to 0 h, there was no substantial difference in the number of downregulated genes between T3 (1104) and CK (1144). In contrast, the number of upregulated genes in T3 (3257) was significantly higher than in CK (1365), suggesting a variation in the biological function of uniconazole induction under cold stress (Fig. 3B, C). The heatmap of top 50 DEG was showed most genes were significantly upregulated at 10 h and 24 h (Fig. S2).

To systematically explore the biological functions of DEGs potentially involved in uniconazole-induced cold tolerance in bananas, we used all DEGs generated by pairwise comparisons in the different treatment groups for KEGG pathway and gene ontology (GO) enrichment analysis. The pathogen interaction (map04626), plant hormone signal transduction (map04075), and MARK signaling pathway-plant (map04016) were generally enriched for DEGs in most comparisons, especially in CK_24h_vs_CK_0h, T3_24h_vs_T3_0h, and T3_24h_vs_T3_10h, and the number of DEGs annotated in the KEGG pathway exceeded 100 (Fig. 3B). The GO enrichment analysis showed that there was a difference in the gene enrichment between the uniconazole-treated and -untreated groups. Unlike the uniconazole treatment groups, the control group, in addition to the common enrichment pathway, mainly focused on the negative regulation of comparisons at different low-temperature treatment times, such as negative regulation of signaling in CK_24h vs. CK_0h and negative regulation of phosphorylation signal transduction in CK_10h vs. CK_0h (Fig. 3C). In contrast, the positive regulation of the response to stimulus was enriched in the uniconazole treatment groups, such as T3_24h_ vs. _T3_0h, T3_24h_ vs. _T3_10, and T3_24h_ vs. _CK_24h. Interestingly, responses to organonitrogen compounds, nitrogen compounds, and chitin were only enriched in T3_24h vs. _CK_24h (Fig. 3C).

### Metabolomic changes associated with uniconazole treatment in bananas

**Fig. 4:**
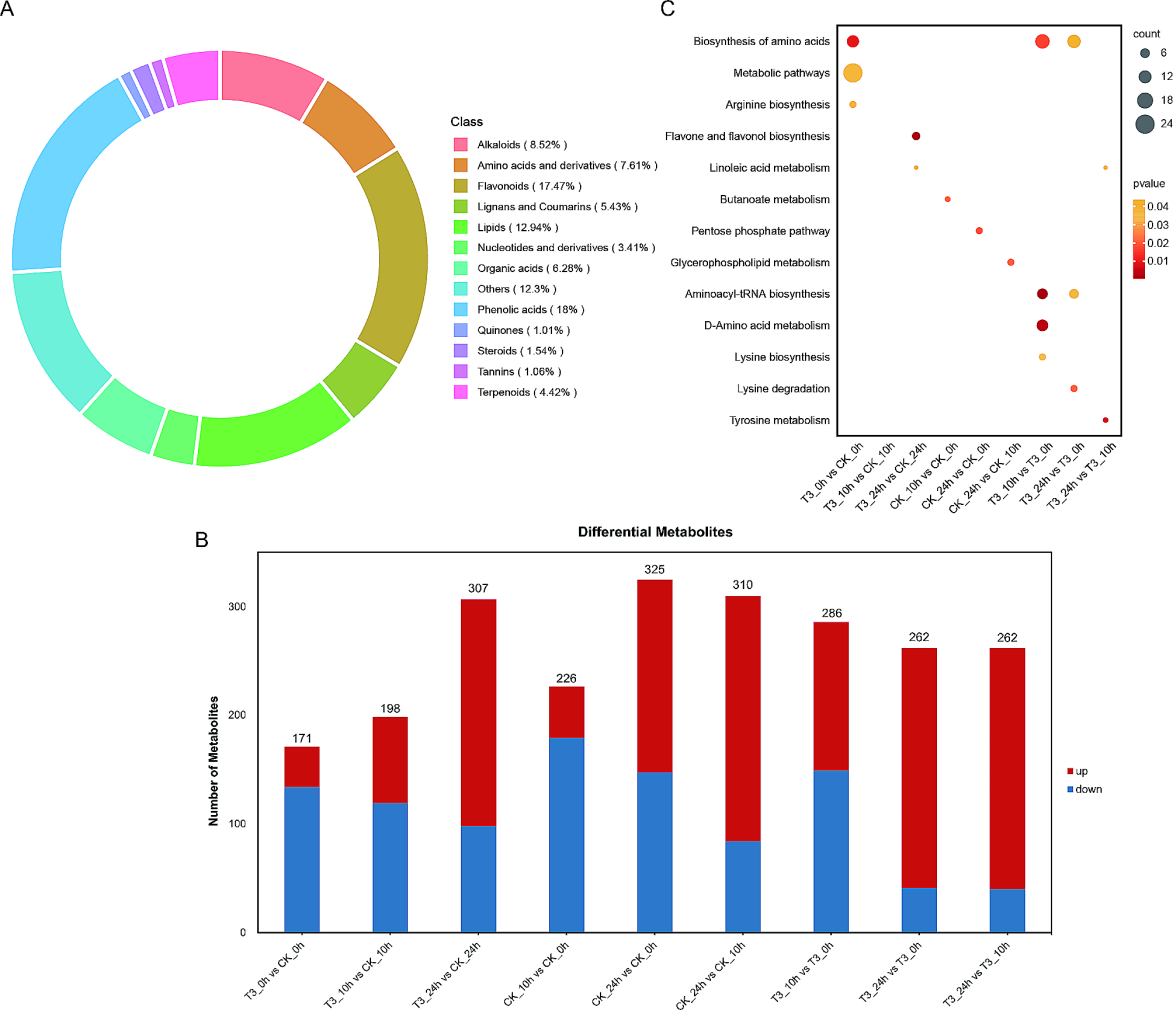
Differentially accumulated metabolites (DAMs) expressed between bananas that received the uniconazole or control treatments induced by cold treatment. **A**: the proportion of detected compound classifications; **B**: the numbers of DAMs between the different groups; **C**: KEGG pathway enrichment analysis of the DAMs. T3 indicate uniconazole treatment with a dosage of 0.2 g. CK indicate control treatment with water

Differential cold tolerance phenotypes suggest that there might be differential concentrations of metabolites in the uniconazole-treated and untreated groups of bananas. Therefore, we performed metabolome analysis using ultra-performance liquid chromatography-mass spectrometry (UPLC-MS) to obtain the metabolome profile of bananas. We detected 1878 metabolites, which were classified into 13 classes: 338 phenolic acids, 328 flavonoids, 243 lipids, 160 alkaloids, 143 amino acids and derivatives, 118 organic acids, 102 lignans and coumarins, 83 terpenoids, 64 nucleotides and derivatives, 29 steroids, 20 tannins, 19 quinones, and 231 others (Table S2 and Fig. 4A). These results suggested that flavonoids, lipids, and phenolic acids were the main metabolites involved in cold tolerance in bananas. Pearson’s correlation coefficient was used to test for correlations between different samples to evaluate the metabolome differences between groups and the variation status among the three replicates (Fig. S3). To explore the differences in metabolites in response to low temperatures, we compared the abundance of the metabolites among the CK and uniconazole treatment groups grown in cold stress conditions (Fig. 4, Fig. S4). As the cold treatment increased in duration, the number of differentially accumulated metabolites (DAMs) increased in uniconazole treatment groups when compared to those in the control group; from 171 in T3_0h vs. CK_0h to 307 in T3_24h vs. CK_24h (Fig. 4B), using threshold values of variable importance in projection (VIP) score ≥ 1 and fold change ≥ 2. We used a differential abundance score to detect global changes in metabolites based on the Kyoto Encyclopedia of Genes and Genomes (KEGG) pathway enrichment analysis. The biosynthesis of amino acid pathways was significantly enriched in T3_0h vs. CK_0h, T3_10h vs. t3_0h, and T3_24h vs. T3_0h (Fig. 4C). In addition, aminoacyl-tRNA biosynthesis, d-amino acid metabolism, and lysine biosynthesis were also significantly enriched in T3_10h when compared to those in T3_0h (Fig. 4C). However, after 24 h of cold treatment, flavone and flavonol biosynthesis and linoleic acid metabolism were enriched in the T3 group when compared to those in the CK group (Fig. 4C).

### Changes in DEGs and DAMs in response to uniconazole treatment in bananas

**Fig. 5:**
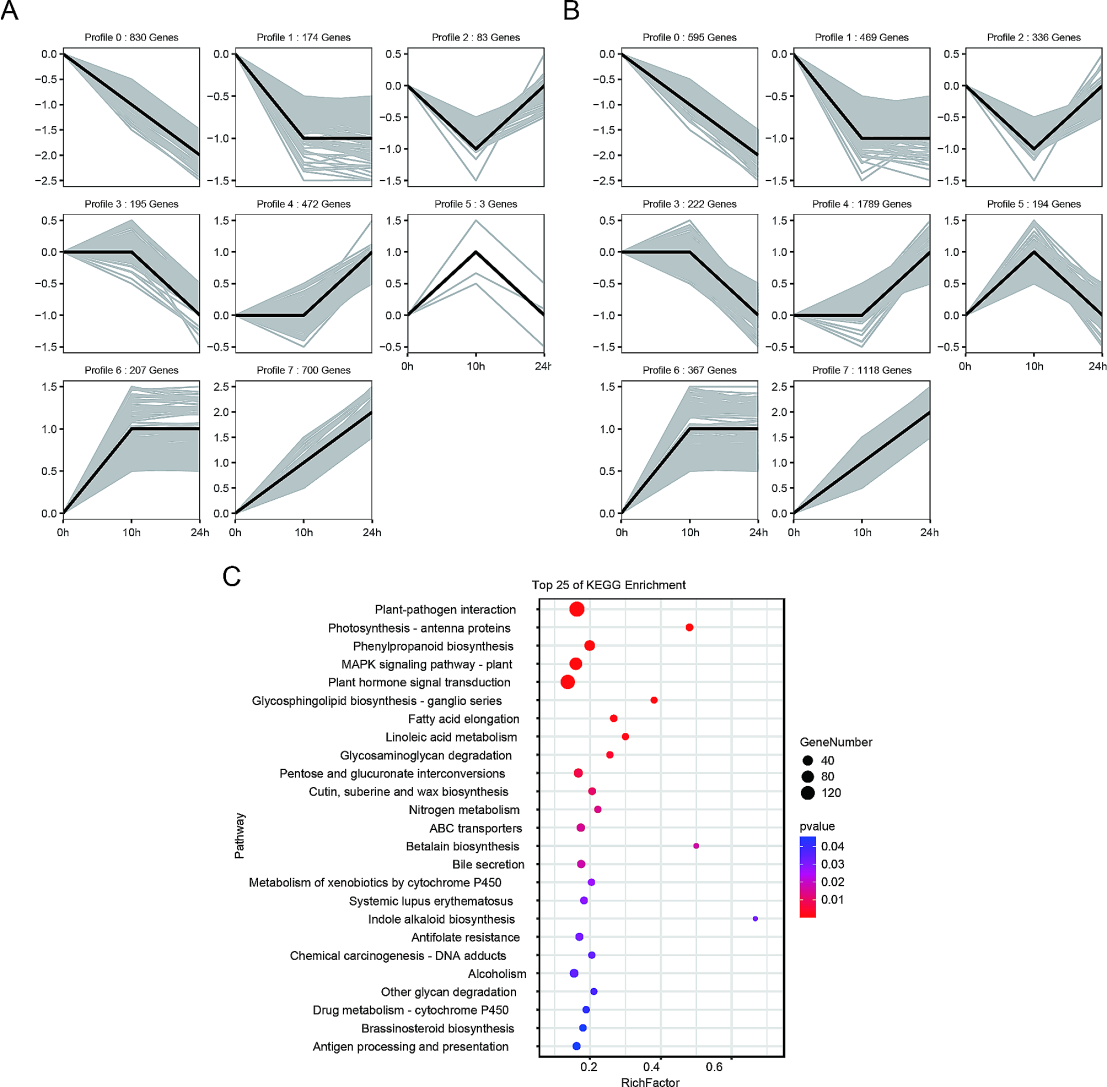
Temporal expression of DEGs response to cold treatment. **A**: the group of CK; **B**: the group of 0.2 g uniconazole treatments; **C**: KEGG pathway enrichment analysis for whose expression profiles showed significant differences

To assess gene expression and accumulated metabolite patterns in response to cold tolerance, the DEGs at different sampling time points (0 h, 10 h, 24 h) after 0.2 g uniconazole treatment (T3) and control (CK) were separated into four types (e.g., down, down_up, up, and up_down) using the STEM algorithm (Fig. 5; Tables S3). Then, we compared with all of the cluster types in CK (Fig. 5A) and T3 (Fig. 5B), then divided all of the genes in the four types into two categories: the same or different types in CK and T3 (Table S3). The same type genes in both groups showed patterns of genetic alteration during the typical course of cold treatment, for example, *PLA2G* (Mba08_g16280) has a same type in CK and T3 (Fig. S5). However, the DEGs reflected the differences in gene expression changes under cold stress after treatment with uniconazole, for example, *LOX2S* (Mba03_g11110) has a different type in CK and T3, down in CK and up_down in T3 (Fig. S5). KEGG pathway enrichment analysis showed that DEGs with different types were mainly clustered in plant-pathogen interactions, phenylpropanoid biosynthesis, the MAPK signaling pathway, plant hormone signal transduction, and linoleic acid metabolism (Fig. 5C).

**Fig. 6:**
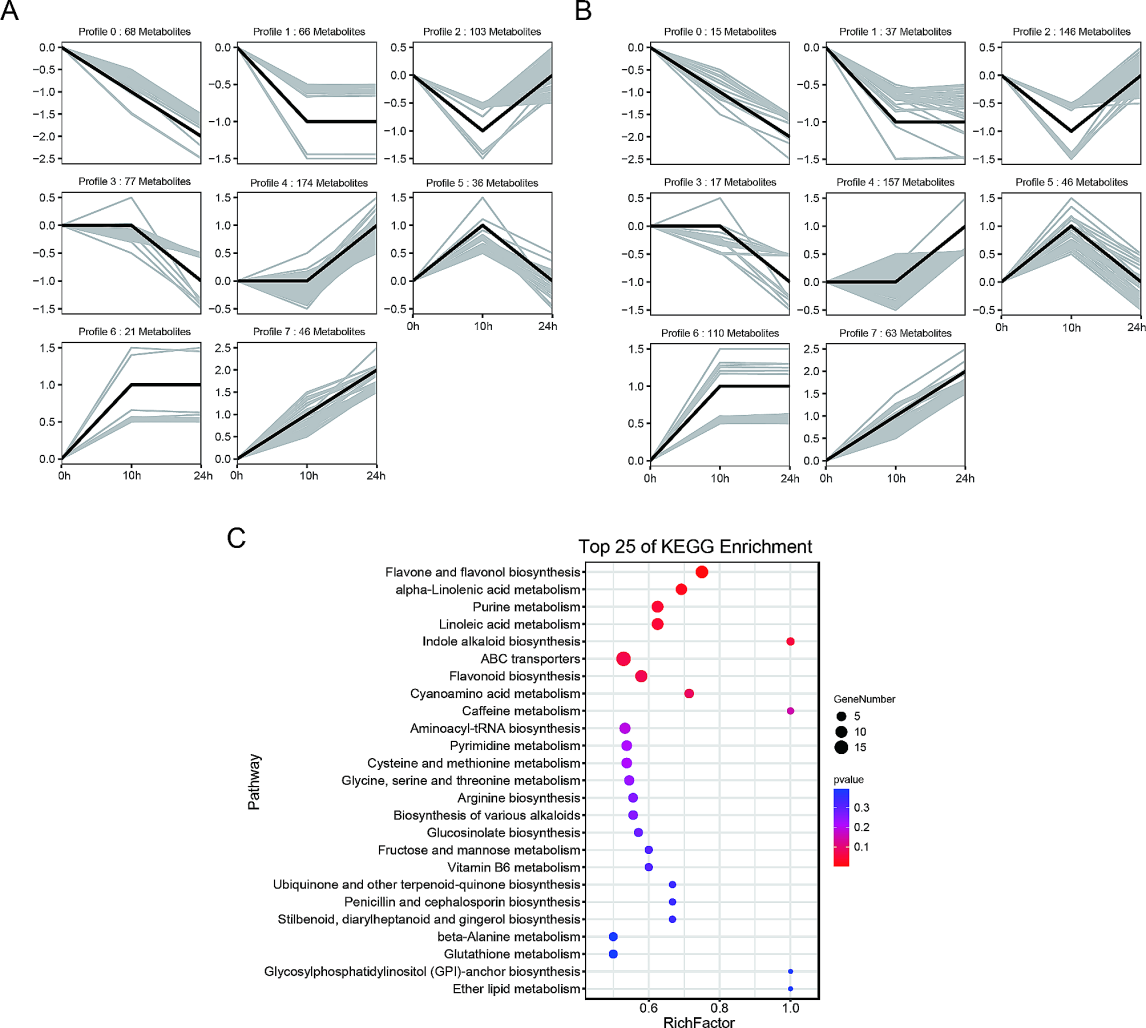
Temporal expression of DAMs response to cold treatment. **A**: the group of CK; **B**: the group of 0.2 g uniconazole treatments; **C**: KEGG pathway enrichment analysis for whose expression profiles showed significant differences

The DAMs were analyzed same as the DEGs (Fig. 6; Table S4), and the KEGG pathway enrichment analysis showed that DAMs with different types were mainly clustered in flavone and flavonol biosynthesis, alpha-linolenic acid metabolism, urine metabolism, linoleic acid metabolism, indole alkaloid biosynthesis, ABC transporters, and flavonoid biosynthesis were enriched DAMs in bananas grown under cold stress after uniconazole treatment (Fig. 6C).

### Key pathways involved in uniconazole-induced cold tolerance banana

**Fig. 7:**
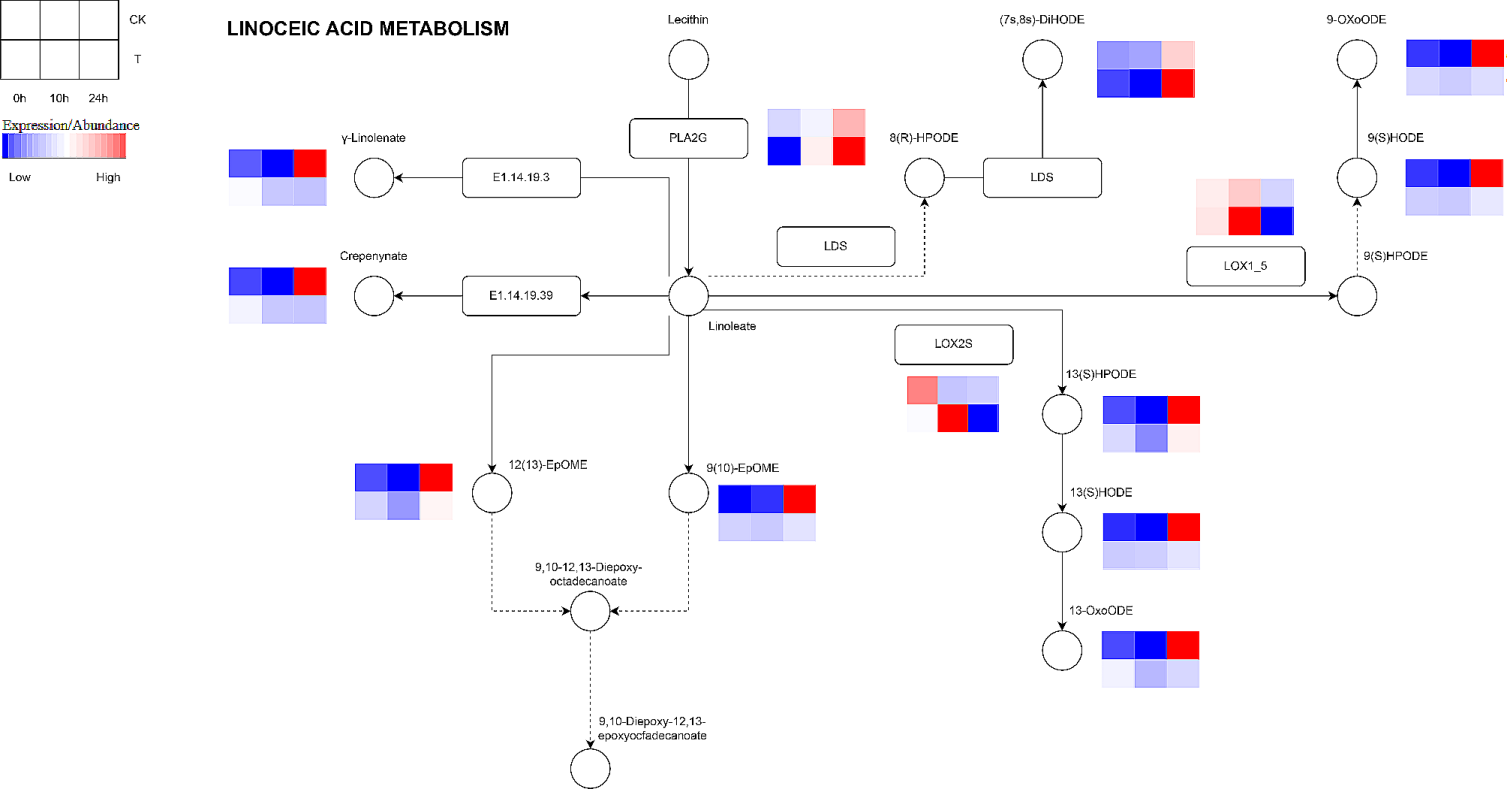
The linoleic acid metabolism pathway was involved in uniconazole-induced cold tolerance in banana. T indicates the uniconazole treatment of 0.2 g. CK indicates the water control treatment

The transcriptional and metabolic analyses of uniconazole-treated bananas grown under cold stress showed that differentially expressed genes and metabolites were coenriched in the linoleic acid metabolism pathway (Fig. 7). In the linoleic acid metabolism pathway, most metabolites, such as γ-Linolenate, Crepenynate,12(13)-EpOME, 9(10)-EpOME, 9-OxoODE, 9(S)HODE, 13(S)HPODE, 13(S)HODE, and 13-OxoODE decreased and then increased in the CK group, while a continuous downward trend was observed in the T3 group. Lox1_5 and LOX2S expression increased and then decreased in the T3 group, whereas they showed a continuous downward trend in the CK group. Furthermore, PLA2G and (7s, 8s)-DiHODE levels showed a continuous upward trend in both groups. The expression of these genes was validated using qRT-PCR (Fig. S5), which showed a high consistency between the transcriptome and qRT-PCR testing.

## Discussion

With an increase in uniconazole treatment concentration, the degree of damage to banana leaves at low temperatures decreased, indicating that uniconazole increased the cold tolerance of banana plants. Low-temperature stress can induce the accumulation of reactive oxygen species (ROS) accumulation. Previous studies have shown that low levels of ROS are necessary for plants to respond to environmental stress [20]. However, excessive accumulation of ROS initiates membrane lipid peroxidation, resulting in increased MDA content. When MDA accumulates, the relative electrical conductivity (REC) increases, resulting in increased membrane permeability and electrolyte exosmosis [21]. MDA is one of the most representative markers used to measure the degree of membrane damage after free radical chain reactions [22]. When intracellular ROS homeostasis is disrupted, free radical-scavenging programs in plant cells are activated in response to the oxidative stress. The antioxidant enzyme system, composed of SOD, POD, and other components, is the most important pathway for free radical removal. Under cold stress conditions, increasing the activities of SOD and POD is conducive to maintaining low levels of ROS in cells to alleviate or defend against low-temperature stress [23, 24]. Compared to the control group, the activities of SOD and POD in the leaves of bananas treated with uniconazole (T2 and T3) significantly increased under low-temperature stress, indicating that the antioxidant system in banana plants was activated. Uniconazole can induce SOD and POD activity in many plants under low-temperature stress [24, 25]; however, in contrast to these previously reported results, the MDA content of bananas was not downregulated by uniconazole when compared to the control group, which may be because it takes some time for the antioxidant system to clear ROS and affect MDA levels. These results indicated that uniconazole can inhibit the ROS accumulation of banana leaves under low-temperature stress by improving the antioxidant efficiency.

Plant signal transduction plays various roles in cold tolerance mechanisms [15]. In plants, ROS not only respond to cold stress but also act as signal transduction molecules that regulate the interaction between plants and microorganisms. Moreover, the balance between their production and clearance is crucial for the adaptive defense response of plants [10, 26]. *Piriformospora indica*, a root endophytic fungus, enhances the cold resistance of bananas by stimulating antioxidant capacity, soluble sugar accumulation, and the expression of cold-responsive genes in leaves [10]. The MAPK cascade responds to ROS, amplifies cold stress signals through phosphorylation, and activates downstream components to promote cold tolerance in plants [27–29]. It is also involved in the signal transduction of various defense responses and plays a key role in the signaling of plant defense against pathogen attack [30, 31]. Based on these studies, plant-pathogen interactions appear to share some mechanisms with cold resistance responses. Zhang et al. (2023) reported that plant-pathogen interactions were significantly enriched in response to cold stress in *Rhododendron chrysanthum* leaves [32]. We hypothesize that the change in DEGs associated with plant-pathogen interactions may also regulate the antioxidant system in bananas in response to cold stress. However, the association between plant-pathogen interactions and cold resistance requires further study.

Plant hormone signal transduction plays an important role in the regulation of cold resistance. For example, auxin signaling is induced in response to cold stress in plantain and promotes glucose decomposition [15, 33]. The soluble sugar content affects cold tolerance in plants [34], while other plant hormones such as ABA, ethylene, and jasmonate have also been reported to regulate plant cold resistance [11, 32, 35]. As a GA inhibitor, uniconazole can alter the content of IAA, ABA, and other hormones in plants [36, 37]. GA induces the degradation of DELLA inhibitory proteins that control key developmental processes and responses to cold stress [38]. Under cold stress, GA signaling is reduced, whereas DELLA proteins accumulate and bind to jasmonate zinc finger inflorescence meristem proteins to activate jasmonic acid (JA) response genes [39]. In addition, JA can promote DELLA accumulation and inhibit the expression of GA biosynthesis genes [40]. The expression of C-repeat-binding factor (CBF)/DREB1 in response to cold stress has been widely reported. The ICE-CBF transcriptional signaling pathway plays a central role in maintaining plant survival and development in response to cold stress [41]. Xu et al. (2022) reported that the overexpression of DREB1F in bananas enhanced their resistance to cold stress [11], and jasmonate has been reported as a possible upstream signal of the ICE-CBF/DREB1 pathway that positively regulates freeze resistance in apples (*Malus domestica*) [42]. Uniconazole may activate JA response genes by inhibiting gibberellin and enhance the cold resistance of bananas. However, owing to the complex regulatory relationships among many plant hormones, the mechanism by which uniconazole regulates plant hormone signal transduction pathways to enhance banana cold resistance requires further study.

The main metabolites involved in banana cold tolerance are flavonoids and lipids, which have been reported to respond to cold stress in various plants. For example, flavonoid accumulation is significantly induced by low temperatures in ginkgo leaves, kale, Arabidopsis, and apples [43–47]. Cold-acclimated plants also often exhibit marked changes in their lipid composition, particularly in their membranes [48]. Analysis of differential metabolites showed that uniconazole affected the flavone and flavonol biosynthesis, and linoleic acid metabolism to enhance cold resistance in banana plants.

Linoleic acid is a liquid unsaturated fatty acid that plays an indispensable role in maintaining membrane integrity under cold stress [49]. Linoleic acid metabolism is significantly enhanced in the leaves of *Canarium album*, rice, and *Rhododendron chrysanthum* in response to cold stress [32, 50, 51]. Linolenic acid, which is converted from linoleic acid, is a precursor of JA synthesis [52]. Omega-3 fatty acid desaturase (FAD3), which catalyzes the conversion of linoleic acid to linolenic acid, promotes the biosynthesis of JA by increasing linolenic acid levels, thus enhancing the cold tolerance of plants [18, 53, 54]. In addition, MYB4 may recruit the histone deacetylase HDA2 to repress the transcription of *FAD3* by affecting its acetylation level. The acetylation level of the *FAD3* promoter is elevated in banana fruits in response to cold stress [49]. And in this study, *PLA2G* in the linoleic acid metabolism pathway was upregulated in both the CK and T3 groups, indicating that the transformation of lecithin into linoleate in banana plants in both groups was enhanced in response to cold stress. Notably, many metabolites downstream of linoleate, including linolenic acid, showed a downward trend in the T3 group when compared to that in the CK group. These results indicated that uniconazole-treated banana plants accumulated more linoleic acid to maintain the integrity of cell membrane and improve cold resistance.

## Conclusion

In summary, under cold stress, uniconazole treatment enhanced the SOD and POD activities of bananas and promoted a series of physiological and metabolic changes, especially changes in the gene expression and production of metabolites involved in flavonoid biosynthesis and linoleic acid metabolism. The DEGs enriched in plant-pathogen interactions and plant hormone signaling may play complex roles in uniconazole-induced cold resistance in banana, while the increased linoleic acid levels may be important for cold resistance in banana leaves by maintaining the integrity of the cell membrane. Based on our results, we propose a uniconazole-induced cold resistance mechanism hypothesis: under cold stress, uniconazole induces the activities of SOD and POD to depress ROS, which may also be regulated by plant-pathogen interactions. Uniconazole-induced cold resistance in banana may also be complexly regulated by plant hormone signal transduction; uniconazole enhances the cold resistance in bananas by affecting the accumulation of flavonoids, as well as promoting the accumulation of linoleic acid, which is conducive to maintaining the integrity of cell membrane and enhancing the cold resistance of plants. These findings provide insights into the mechanisms underlying the inducible cold resistance in banana and may help expand the production of this important economic crop in the future.

## Materials and methods

### Plant materials and treatment

The experiments were performed at the Libang Scientific Base of the Guangxi Academy of Agricultural Sciences, located in Futang Town, Wu Ming, Nanning, Guangxi, China. The banana cultivar ‘Guijiao No. 9’ was used, with seeds planted in March 2023. Uniconazole wettable powder (5%, Sichuan Runer Technology, China) was applied to the plants when they had 7–8 leaves. After diluting the powder to create concentration gradients, 50 mL solutions were sprayed along the base of the pseudostem, yielding gradient dosages of 0.05, 0.1, and 0.2 g/plant. Control plants received the same volume of water alone. The uniconazole-treated and control groups each included 20 plants that were grown at room temperature for 10 days and then the banana seedlings were moved to 4 ± 1 °C for cold treatment. Samples were taken from the final unfolded leaf at 0 h, 10 h, and 24 h after cold treatment. The samples were frozen in liquid nitrogen and then stored at -80 °C for analysis. Three separate biological replicates were performed for each experiment.

### The measurement of physiological and biochemical indices

Superoxide dismutase (SOD) activity was measured using the xanthine oxidase method, which is based on the production of O^2−^ (Suzhou Keming Biotechnology Co., Ltd, Suzhou, China). Peroxidase (POD) activity was examined by measuring the H_2_O_2_ oxidation of specific substrates (Suzhou Keming Biotechnology Co., Ltd, Suzhou, China). The soluble proteins were quantified using the reduction of Cu^2+^ to Cu^1+^ in an alkaline environment (Nanning Guotuo Biotechnology Co., Ltd., Nanning, China). Malondialdehyde (MDA) was identified by producing a Thompson result upon condensation with thiobarbituric acid (Suzhou Keming Biotechnology Co., Ltd., Suzhou, China). Proline (PRO) is detected by its reaction with an acidic ninhydrin solution to generate a red solution (Beijing Solarbio Science & Technology Co., Ltd., Beijing, China). SOD, POD, MDA, PRO, and soluble protein were determined by measuring sample absorbance at 560, 470, 532, 520, and 562 nm, respectively.

### RNA extraction and RNA-Seq

Total RNA was extracted from banana seedlings, with three biological replicates for each treatment using a Qiagen RNeasy Plant Kit (Hilden, Germany) following the manufacturer’s protocol. DNA contamination and the quality, concentration, and integrity of the total RNA were measured using agarose gel electrophoresis, Nanodrop 2000, and Agilent 2100 BioAnalyzer, respectively. RNA-seq libraries were prepared using the Illumina TruSeq RNA Sample Prep Kit, following the manufacturer’s instructions, and the quality of the library was determined using a Qubit2.0 and qPCR. The cDNA library products that passed the quality tests were sequenced using the Illumina NovaSeq platform.

### Transcriptome analysis

To obtain high-quality clean reads, read sets were subjected to adapter removal and quality analysis using Fastp [55]. Read sets with N content exceeding 10% of the number of read bases were considered low-quality sequences and were filtered out of the data. HISAT2 (v2.1.0) was used for positional information in the reference genome, sequence characteristic information specific to sequencing samples, and the sequence alignment of clean reads with the banana reference genome (NCBI accession No. GCF_000313855.2) [56].

The unigenes were functionally annotated and classified using public databases, including nr protein, Swiss-Prot, KEGG, TREMBL, Gene Ontology (GO), and Clusters of Orthologous Groups of Proteins (COG) using BLAST software. The expression value of each unigene was normalized to fragments per kilobase of transcript per million fragment-mapped reads (FPKM). Pearson’s correlation coefficients between samples were computed based on the gene expression levels. To identify differentially expressed genes, we used the DEseq2 package (1.22.2) in R to analyze unstandardized read count data between two samples based on a false discovery rate (FDR) < 0.05, and absolute log2 FC ≥ 1.

### Metabolite extraction and LC-MS/MS analysis

The metabolite extracts were freeze-dried under vacuum and ground into a powder (30 Hz, 1.5 min). Powdered plant tissues (50 mg) were extracted using 1.2 mL precooled 70% methanol. Vortex oscillation was conducted every 30 min for 30 s six times. The solutions were centrifuged at 12,000 rpm for 3 min before the supernatant was transferred to a new 1.5-mL Eppendorf tube. The insoluble fraction was filtered using a 0.22-µm microporous membrane and stored in a sample vial for UPLC-MS/MS analysis.

Ultra-performance liquid chromatography (UPLC) was performed using a ExionLC™ AD and Tandem mass spectrometry (MS/MS) analysis was conducted using an Applied Biosystems 6500 QTRAP with an Agilent SB-C18 1.8 μm, 2.1 × 100 mm column. The injection volume was 2 µL and a binary separation gradient was applied at a flow rate of 0.35 mL/min: 0 min, isocratic 95% A (ultra-pure water with 0.1% formic acid), 5% B (acetonitrile with 0.1% formic acid); 0 to 9 min, linear gradient to 95% B; 9 to 10 min, isocratic 95% B; 10 to 11.1 min, linear gradient to 5% B. The main conditions for mass spectrometry were electrospray ionization (ESI) source temperature 500 °C; ion spray voltage (IS) 5500 V (positive ion mode)/-4500 V (negative ion mode); ion-source gas I (GSI), gas II (GSII), and curtain gas (CUR) were set to 50, 60, and 25 psi, respectively. Collision-induced ionization parameters were set to “high.” The metabolites were quantified by multiple reaction monitoring (MRM) analysis using triple quadrupole mass spectrometry based on a self-established software database (MWDB) [57, 58].

### Metabolome analysis

Metabolomic analysis was performed using Analyst 1.6.3. Pearson’s correlation coefficient was calculated using the cor function in R software. To identify DAMs, we implemented orthogonal partial least squares discriminant analysis (OPLS-DA) using MetaboAnalystR, according to the following thresholds: variable importance in projection (VIP) score ≥ 1 and absolute log2 FC ≥ 1. Pathway enrichment analysis of the identified metabolites was performed by mapping them to the Kyoto Encyclopedia of Genes and Genomes (KEGG) database. The significant pathways of the DAMs were determined using the *P*-values obtained from the hypergeometric test.

### Temporal analysis

Short time-series expression miner (STEM) software can process short time series data for clustering and statistical biological results using exclusive approaches to integrate them with KEGG databases. We used the STEM algorithm with default parameters to analyze trends in changes in the gene expression profiles of bananas during cold tolerance. The DEGs and DAMs were clustered according to their *P*-values. Clustered profiles with *P* ≤ 0.05 were considered differentially expressed. Genes within the selected clusters were enriched in KEGG pathways for functional annotation using the hypergeometric distribution test. Functional items of each selected cluster with Q-values ≤ 0.05 were retained.

### qRT-PCR analysis

Quantitative reverse transcription PCR (qRT-PCR) was conducted to authenticate the expression of pivotal genes. RNA isolated from the six groups banana were transcribed into cDNA utilizing the MonScript™ RTIII (Monad). qRT-PCR was executed on the Applied Biosystems 7500 Real-Time PCR System with specific primers (Table S5). The relative mRNA levels of detected genes were calculated according to the 2^(−ΔΔCt)^ algorithm and *β*-Actin gene was served as an internal control [59].

### Statistical analysis

Three replicates were analyzed for each sample type. Pearson correlation coefficients were calculated between the abundance of DAMs and DEGs from metabolomic profiling and between the relative expression from RNA-seq across stages using R (v3.6.3). Data are expressed as the means ± SE, the differences among three or more groups were analyzed using Tukey’s test. The results were considered statistically significant if the *p* value < 0.05.

### Electronic supplementary material

Below is the link to the electronic supplementary material.

Supplementary Material 1

Supplementary Material 2

## Data Availability

The datasets used and/or analyses during the current study are available in the NCBI Bioproject repository, [PRJNA1055711].
