# Supplementary material for: Discovery of gene regulation mechanisms associated with uniconazole-induced cold tolerance in banana using integrated transcriptome and metabolome analysis
Source: BMC Plant Biol. 2024 Apr 26;24:342. doi: 10.1186/s12870-024-05027-2 (PMC11046889; doi:10.1186/s12870-024-05027-2)
Supplement: Supplementary file 1 — Supplementary Material 1 [file 12870_2024_5027_MOESM1_ESM.pptx]

## Slide 1
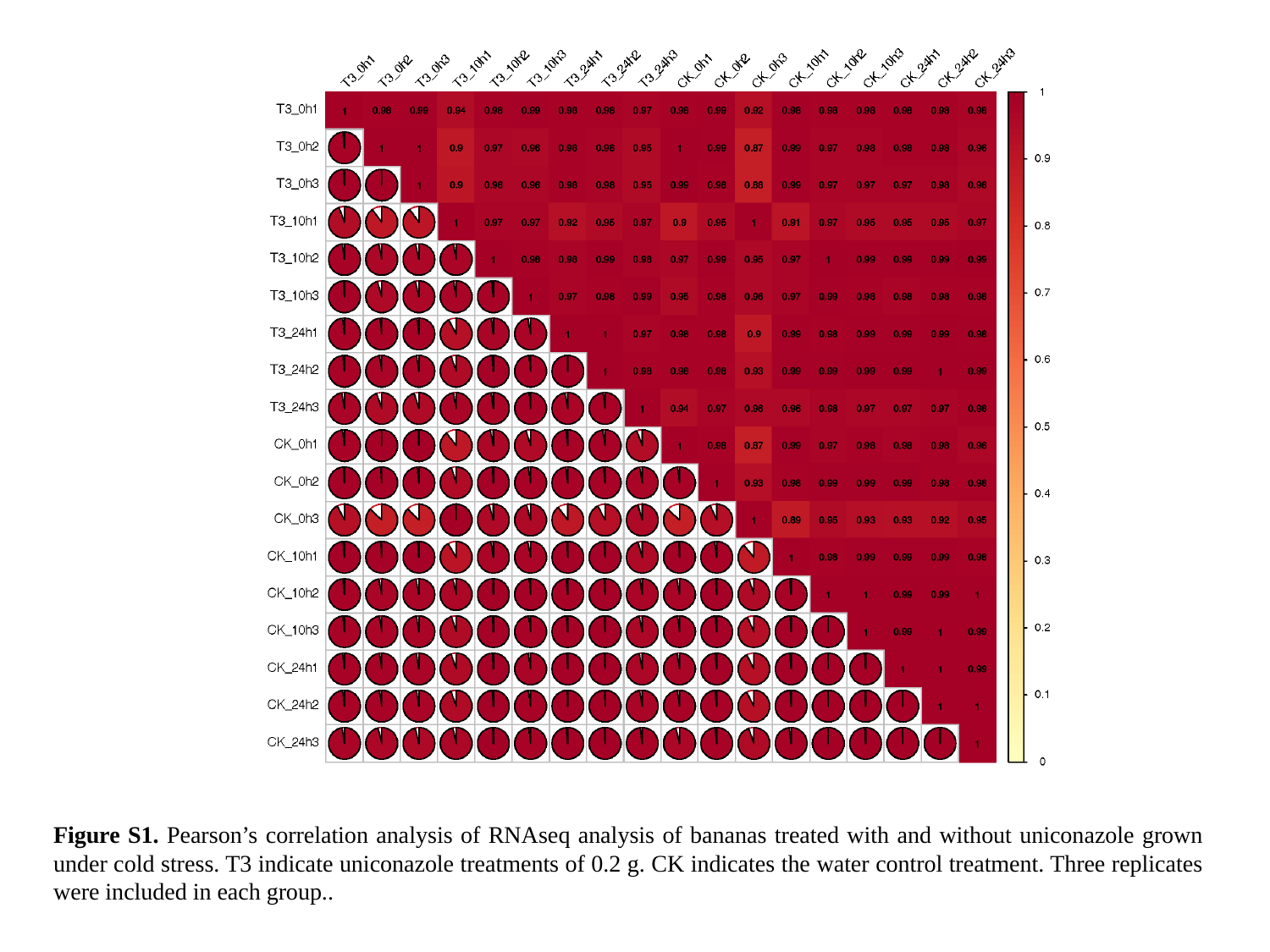

Figure S1. Pearson’s correlation analysis of RNAseq analysis of bananas treated with and without uniconazole grown under cold stress. T3 indicate uniconazole treatments of 0.2 g. CK indicates the water control treatment. Three replicates were included in each group..

## Slide 2
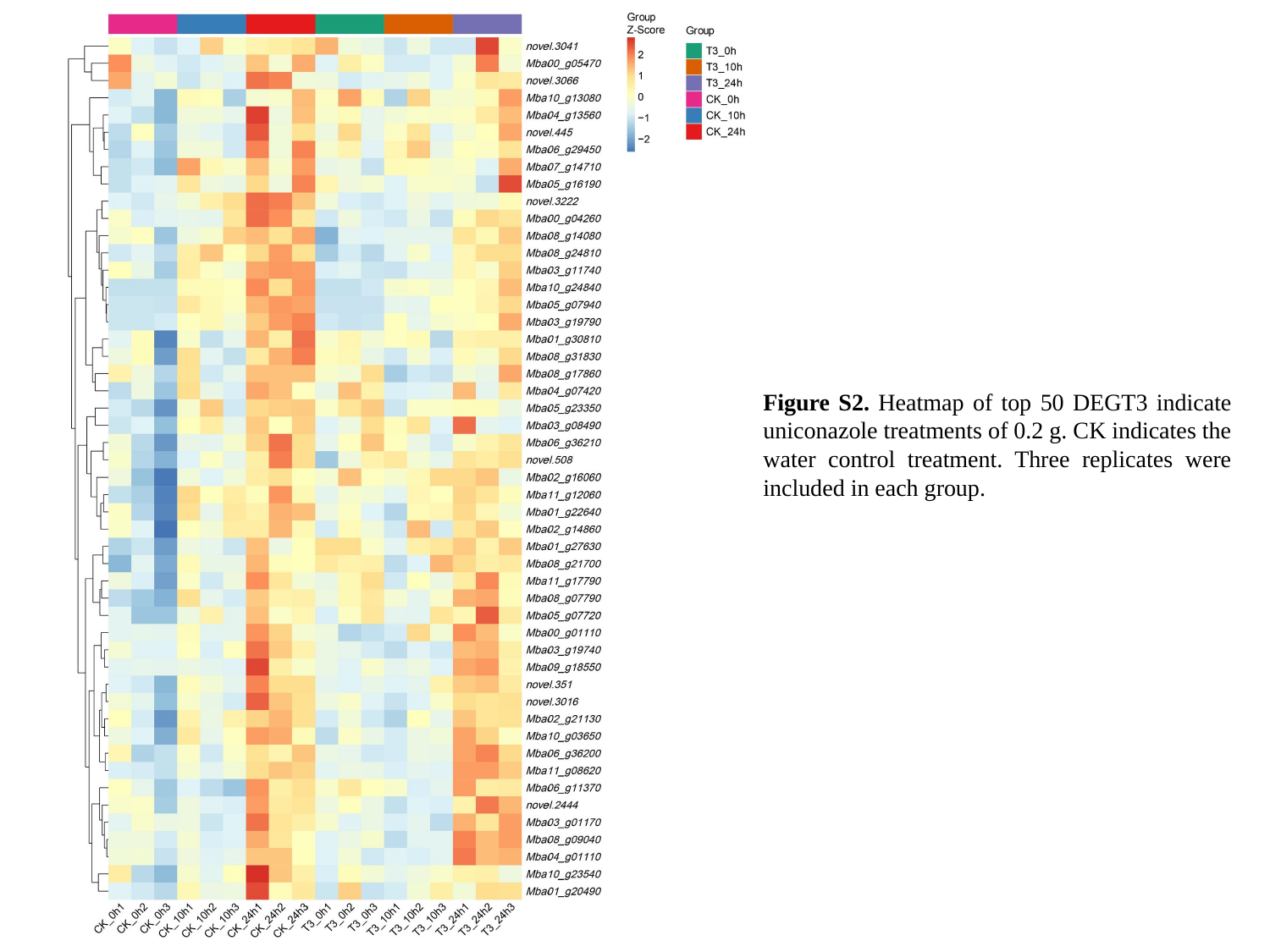

Figure S2. Heatmap of top 50 DEGT3 indicate uniconazole treatments of 0.2 g. CK indicates the water control treatment. Three replicates were included in each group.

## Slide 3
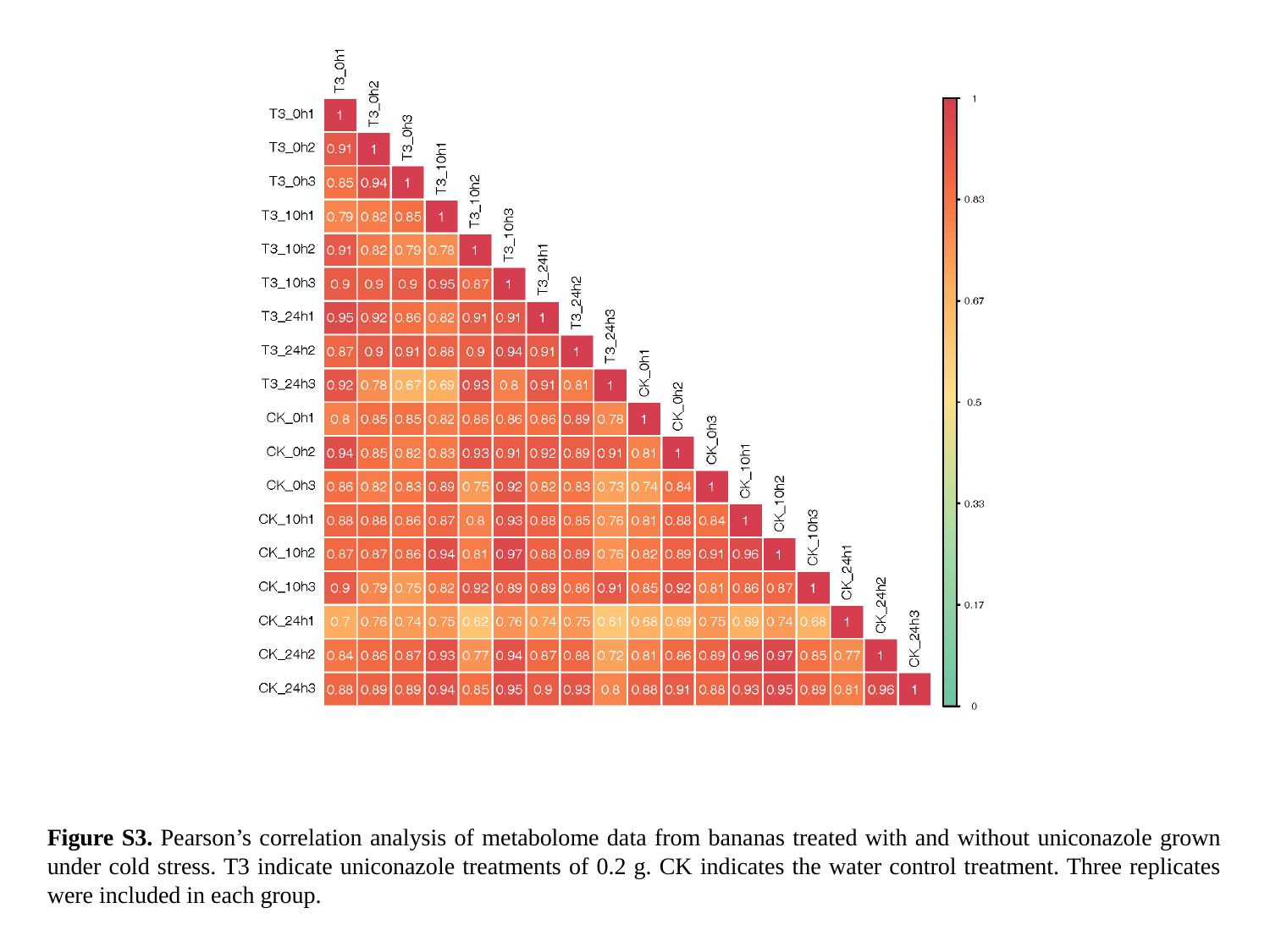

Figure S3. Pearson’s correlation analysis of metabolome data from bananas treated with and without uniconazole grown under cold stress. T3 indicate uniconazole treatments of 0.2 g. CK indicates the water control treatment. Three replicates were included in each group.

## Slide 4
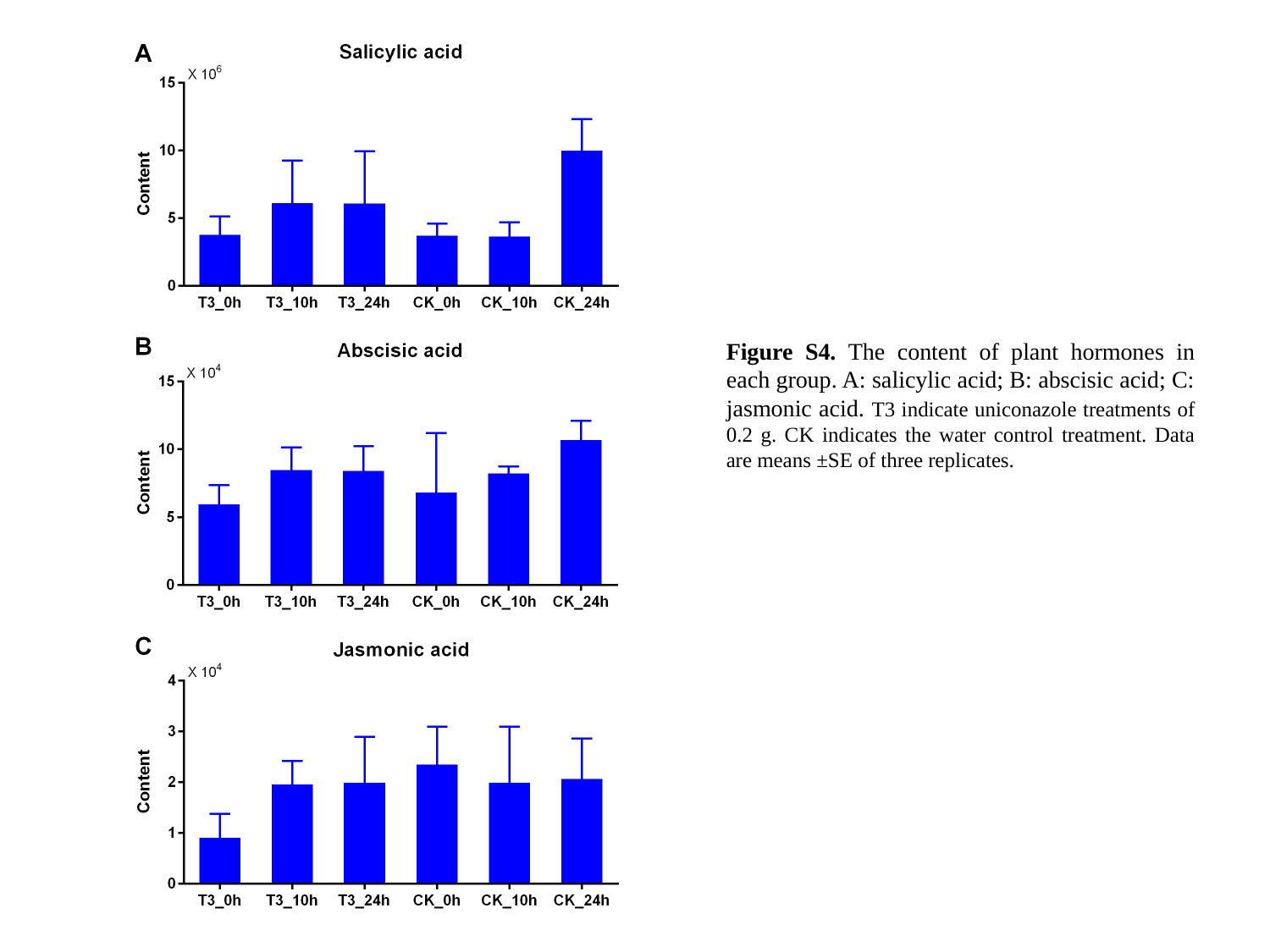

Figure S4. The content of plant hormones in each group. A: salicylic acid; B: abscisic acid; C: jasmonic acid. T3 indicate uniconazole treatments of 0.2 g. CK indicates the water control treatment. Data are means ±SE of three replicates.

## Slide 5
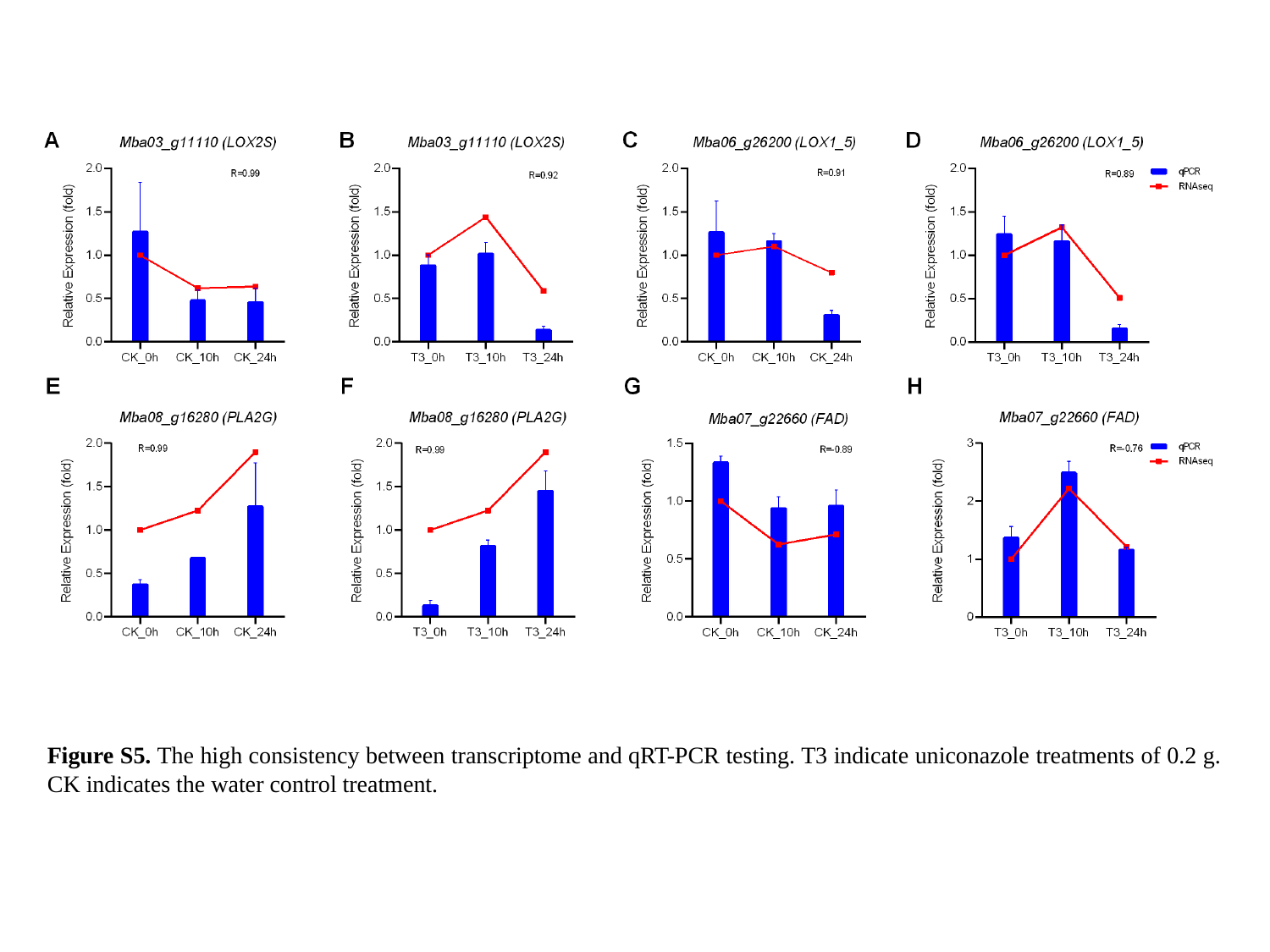

Figure S5. The high consistency between transcriptome and qRT-PCR testing. T3 indicate uniconazole treatments of 0.2 g. CK indicates the water control treatment.
